# Assessment of the Relationship Between Orthorexia Nervosa, Eating Attitudes and Behaviors, and Obsessive–Compulsive Disorder in University Students

**DOI:** 10.1002/brb3.70583

**Published:** 2025-07-03

**Authors:** Mehtap Gomleksiz, Ezgi Yarasir

**Affiliations:** ^1^ School of Medicine, Department of Family Medicine Firat University Elazig Türkiye; ^2^ Department of Therapy and Rehabilitation, Vocational School of Health Services Firat University Elazig Turkey

**Keywords:** eating behavior, eating disorder, obsessive–compulsive disorder, orthorexia nervosa

## Abstract

**Background:**

This study aimed to determine the psychosocial risk factors for orthorexia nervosa (ON) in university students and to evaluate the relationship between eating attitudes and behaviors and obsessive–compulsive disorder (OCD).

**Methods:**

A cross‐sectional study was conducted with 882 university students in an eastern Turkish province from October to December 2023. Data for the study were collected using a sociodemographic survey form, the Orthorexia Nervosa Test (ORTO‐15), the Eating Attitude Test (EAT‐40), and the Obsessive–Compulsive Inventory‐Revised (OCI‐R) assessments. Additionally, an online survey was administered to the participants using Google Forms. The collected data were analyzed using the SPSS 22.0 statistical software program.

**Results:**

The participants had a mean age of 20.9 ± 3.0 years (range: 17–45), and 73.1% (*n* = 645) were female. Orthorexic tendencies were observed in 14.7% of the participants, impaired eating behavior in 15.6%, and a risk of OCD in 58.7%. The mean ORTO‐15 score for the participants was 37.3 ± 3.6, the mean EAT‐40 score was 20.0 ± 15.5, and the mean OCI‐R score was 25.9 ± 15.0. ORTO‐15 scores were significantly higher among participants in their sixth year of study and those enrolled in evening education programs (*p* < 0.05). EAT‐40 scores were significantly higher in students aged 20–22, those with poor socioeconomic status, and smokers (*p* < 0.05). OCI‐R scores were notably higher in participants who lived in dormitories during their studies and those who had quit smoking (*p* < 0.05). While ORTO‐15 scores were negatively correlated with EAT‐40 scores (*p* < 0.05), a weak positive correlation was observed between OCI‐R and EAT‐40 scores (*p* < 0.05).

**Conclusion:**

Although ON, OCD, and eating disorders seem to be different disorders, they both have similar characteristics. Further studies are needed to investigate possible risk factors of comorbidity.

## Graphical abstract

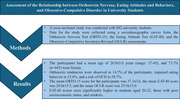

## 1 Introduction

Orthorexia nervosa (ON) was initially defined by the doctor Steven Bratman as a disorder marked by a pathological fixation with healthy or “pure” food and appropriate nutrition. (Pontillo et al. 2022). In daily life, people with ON spend a lot of time organizing and cooking nutritious meals using “pure food” and avoid eating with others. Notably, they care excessively about the ingredients used in food preparation and cut out items they deem impure due to the presence of artificial compounds or pesticides (Pontillo et al. 2022). ON is not included in the Diagnostic and Statistical Manual of Mental Disorders (DSM)‐V classifications (Niedzielski and Kaźmierczak‐Wojtaś 2021). As a result, there are no recognized and confirmed diagnostic standards for the condition. The prevalence of ON in university students varied between 6.9% and 88.76% (Niedzielski and Kaźmierczak‐Wojtaś 2021). People with ON exhibit extreme control over their meals and spend significant time on this behavior (Donini et al. 2004). ON leads to nutritional deficiencies, weight loss, and other medical issues from dietary choices and unbalanced nutrition. It can cause severe emotional distress due to consuming foods perceived as unhealthy and limit social functioning due to obsessive thoughts about healthy eating (Dunn and Bratman 2016).

One of the primary discussions regarding ON is whether it is connected to the spectrum of eating disorders (ED) or obsessive–compulsive disorder (OCD). Recent evaluations suggest that ON shares similarities with a wide range of ED, emphasizing the significance of eating in an individual's life, the close relationship between food and self‐esteem, and the social and health implications involved (Cena et al. 2019). However, ON and OCD appear to have similar cognitive rigidity, perfectionist qualities, healthy food‐related obsessions and compulsions, and ritualistic behavior linked to meal preparation, consumption, and buying. ON obsessions are viewed as normal and adequate (Mathieu 2005), in contrast to OCD obsessions, which are typically seen as egodystonic and cause patients great discomfort and a desire to change (Scarff 2017). Patients with OCD tend to report distress from compulsive behavior and a desire to change, thus demonstrating insight into their illness (Scarff 2017). Both ED and ON are characterized by a lack of pleasure associated with eating and a need to control food intake as a means to improve their self‐esteem or self‐actualization, giving them a sense of control over their own lives (Parra Fernández et al. 2018). People with orthorexia are focused on eating healthy and pure foods, obsessed with quality, while people with anorexia and/or bulimia are more concerned with the quantity rather than the quality of the food they eat (Varga et al. 2014). Although weight loss in anorexia nervosa and feeling healthy in ON are related, it is stated that similar social and psychological consequences can be found in both disorders (Varga et al. 2014).

Youth is a period when long‐lasting habits are formed. Unhealthy eating attitudes are identified as one of the dangerous behaviors observed during this period (Kann et al. 2000). In addition, socioeconomic status, presence of chronic disease, and habits such as smoking have been stated as factors that increase the risk of ED (Thomas et al. 2002; Maalouf et al. 2023; Harrison et al. 2020). Early detection of ON is crucial to prevent its development in other EDs and to facilitate the creation of appropriate therapies (McComb and Mills 2019). Approximately 90% of eating problems occur in persons who are 25 years old or younger. During college and university years, people are at a key stage where they face increased susceptibility to hazardous changes in eating habits, according to Agopyan et al. (2019). Studies have shown that individuals with ON may experience negative effects on their academic performance due to strict dietary restrictions that can lead to nutrient deficiencies and impaired cognitive function (Parra Fernández et al. 2018). In the study conducted in the medical student population, 1.6% of the participants reported having an eating disorder that affected their academic performance, and 29.1% stated that it was one of the most threatening health barriers to their academic performance (Kernan et al. 2008). There are studies that show that treating college students with ED may be associated with academic performance (Claydon and Zullig 2020). Adapting to new social roles, loss of family support due to moving away from home, stress of choosing a career, living with people from different sociocultural backgrounds, and economic difficulties are some of the difficulties that university students face. It has been reported that such stressful situations can affect students' mental health and lead to symptoms of depression and ED (Trindade et al. 2019). Distress, academic self‐efficacy, and ON are interrelated psychological variables that can influence and reinforce each other. The mediating effect of psychological distress suggests that it may act as a mediating factor between ON and academic self‐efficacy (Barakat et al. 2024). The collaboration between educational institutions and mental health professionals is important to offer help and resources to students struggling with academic stress (Barbayannis et al. 2022). It may help reduce the likelihood of resorting to unhealthy coping mechanisms such as ON. It is known that academic success and mental well‐being are interconnected and need to be addressed holistically to promote positive behaviors and outcomes (Barakat et al. 2024).

The aim of this study is to focus on whether individual characteristics such as sociodemographic characteristics, health, nutrition, and physical activity behaviors create differences in terms of ON, OCD, and EAT.

## 2 Methods

The population of this cross‐sectional study consisted of all students studying at a university's medical faculty (*N* = 1329), education faculty (*N* = 2250), and vocational school of health services (*N* = 1607) in a city with a population of 600 thousand in the east of Turkey.

The sample size was determined using the formula *n* = *Nt* ^2^ *pq*/*d* ^2^(*N − *1) + *t* ^2^ *pq*.

*N*: Number of individuals in the population (5186)

*n*: Number of individuals to be sampled (862)

*p*: The frequency (probability) of the event analyzed (41.2%) (incidence of OCD) (Arad et al. 2023)

*q*: Frequency of nonoccurrence of the examined event (58.8%)

*d*: Indicates the desired ± deviation (3% = 0.03) according to the frequency of the event.

*t*: 1.96

The minimum sample size was found to be 862. Inclusion criteria were as follows: being a student of the Faculty of Medicine/Faculty of Education/Vocational School of Health Services and being voluntary and willing to participate in the study. The data collection was completed between October and December 2023. A total of 882 participants were included in the study. The questionnaire was administered using an online survey method through Google Forms, with an average completion time of 15–20 min.

### 2.1 Data Collection Instruments

#### 2.1.1 Sociodemographic Form

The study used a questionnaire consisting of four sections as a data collection tool. In the first part of the questionnaire, there are 25 questions prepared by the researchers based on the literature, evaluating the sociodemographic characteristics of the individuals related to age, gender, height, weight, educational status, chronic disease, smoking and alcohol use status, and eating habits.

#### 2.1.2 Orthorexia Nervosa Test

The Orthorexia Nervosa Test (ORTO‐15) is a 15‐item self‐assessment scale developed by Donini in 2004 by adapting Bratman's short questionnaire and designed to assess the tendency to ON (Bratman and Knight 2000; Donini et al. 2004). Items 2, 5, 8, and 9 are reverse‐scored. Each item is scored on a scale of 1 to 4 points. In Turkey, the validity and reliability of the ORTO‐15 test were performed by Arusoğlu (2006). The scale consists of 15 items answered as *always*, *often*, *sometimes*, and *never* in order to assess individuals’ obsessive attitudes towards choosing, purchasing, preparing, and consuming food. A minimum of 15 and a maximum of 60 points are obtained from the scale. Those with healthy eating habits (orthorectics) score lower on this scale. The cut‐off score of the scale is 33, and 33 points and below are classified as orthorectic tendency. The higher the score, the closer the eating behavior is to normal from hypersensitivity. Responses indicating ON symptoms received 1 point, while responses defining adequate eating behaviors received 4 points. The Cronbach's alpha value of the scale is 0.44 (Arusoğlu 2006).

#### 2.1.3 Eating Attitude Test

The third part of the questionnaire included the Eating Attitude Test (EAT‐40). The EAT‐40 is a self‐assessment scale developed by Garner and Garfinkel (1979). The items are in a six‐point Likert‐type scale with *always*, *very often*, *often*, *sometimes*, *rarely*, and *never* options and consist of 40 questions. The cut‐off score is 30, and scores of 30 and above are indicative of disordered eating. Turkish validity and reliability studies were conducted by Savaşır and Erol (1989). The Turkish version of the EAT‐40 measures disordered eating attitudes and behaviors. An increase in score is associated with an increased risk of eating behavior disorder. The Cronbach's alpha value of the scale is 0.70 (Savaşır and Erol 1989).

#### 2.1.4 Obsessive–Compulsive Inventory‐Revised

The Obsessive–Compulsive Inventory‐Revised (OCI‐R) is a self‐report instrument developed by Foa et al. (2002). It consists of 18 self‐report items to assess the presence and severity of obsessive–compulsive symptoms, rated on a five‐point scale ranging from 0 (*not at all*) to 4 (*extremely*). The OCI‐R yields scores across six factors: washing, checking, obsessions, mental neutralizing, ordering, and hoarding. Turkish validity and reliability were performed by Aydin et al. (2014). Those who scored a total of 21 and above on the scale were considered to have a high probability of having OCD. The Cronbach's alpha value of the Turkish version of OCI‐R is 0.89. (Aydin et al. 2014).

### 2.2 Statistical Analysis

SPSS 22.0 was used for the analysis of the data from the study. Mann–Whitney *U*, Kruskal–Wallis, and Spearman correlation analyses were used because the data did not have a normal distribution based on the Kolmogorov–Smirnov test. In groups where there was a difference, the Bonferroni test was used to identify the source of the difference. The relationship between various characteristics of the sample group (gender, faculty, class, etc.) and ORTO‐15, EAT‐40, and OCI scores was analyzed. The corrected *p* value was used for statistical significance in multiple group comparisons. Means were given with standard deviation (mean ± SD), and *p* < 0.05 was considered statistically significant.

## 3 Results

The mean age of the participants was 20.9 ± 3.0 years (min: 17, max: 45), and 73.1% (*n* = 645) were female. 55.0% of the students reside with their families during their education. The participants’ sociodemographic characteristics are given in Table 1.

**TABLE 1:** Distribution of participants according to sociodemographic characteristics, health, nutrition, and physical activity characteristics.

Sociodemographic characteristics (*n* = 882)	*n*	%
Gender		
Female	645	73.1
Male	237	26.9
Age		
17–19 age	294	33.3
20–22 age	416	47.2
23 years and over	172	19.5
Departments		
Faculty of medicine	279	31.6
Vocational school of health services	385	43.7
Faculty of education	218	24.7
Grade		
First grade	335	38.0
Second grade	273	31.0
Third grade	116	13.2
Fourth grade	55	6.2
Fifth grade	30	3.4
Sixth grade	73	8.2
Type of education		
Formal education	784	88.9
Secondary education	98	11.1
Mother's education level		
Illiterate	141	16.0
Literate primary school	434	49.2
Middle school–high school	217	24.6
University and above	90	10.2
Father's education level		
Illiterate	19	2.2
Literate primary school	287	32.5
Middle school–high school	345	39.1
University and above	231	26.2
Perception of socioeconomic situation		
Good	108	12.2
Middle	697	79.1
Poor	77	8.7
Place of residence during education		
With friends—Alone	76	8.6
With family	485	55.0
In the dormitory	321	36.4
Place of residence before university		
City	637	72.2
Town	133	15.1
Village	112	12.7
Smoking		
Yes	133	15.1
Quit	55	6.2
No	694	78.7
Alcohol use		
Yes	60	6.8
Quit	38	4.3
No	784	88.9
Chronic disease condition		
Yes	83	9.4
No	799	90.6
Medication		
Yes	122	13.8
No	760	86.2
Perception of health condition		
Good	443	50.2
Middle	424	48.1
Poor	15	1.7
Regular breakfast		
Yes	479	54.3
No	403	45.7
Perception of nutritional status		
Very good	29	3.3
Good	621	70.4
Bad	211	23.9
Very bad	21	2.4
The source of information about nutrition^a^		
Internet	723	81.9
Family	484	54.8
Doctor	312	35.3
Friends	296	33.5
Books, scientific publications	225	25.5
Dietitian	189	21.4
Television	181	20.5
Magazine	23	2.6
Newspaper	22	2.5
Dieting status		
Yes	68	7.7
No	814	92.3
Type of diet (*n* = 68)		
Calorie restriction diet	39	4.4
Gluten diet	9	1.0
Vegetarian diet	7	0.8
Calorie increase diet (weight gain)	4	0.5
Protein‐intensive diet	3	0.3
Other (intermittent diet, FODMAP diet, raw food diet)	3	0.3
Chronic disease diet (such as DM, HT)	2	0.2
Regular physical activity		
Yes	239	27.1
No	643	72.9

*Note*: Percentages are based on “*n*.”

^a^ One person responded to more than one option.

Of the participants, 14.5% (*n* = 128) were underweight, 66.8% (*n* = 589) were normal weight, 16.7% (*n* = 147) were overweight, and 2.0% (*n* = 18) were obese. Nutritional status was considered good by 70.4% of participants. Individuals accessed information about nutrition through the internet, family, and doctor, respectively (Table 1).

Orthorexic tendencies were detected in 14.7% of the participants, impaired eating behavior in 15.6%, and symptoms of OCD in 58.7%. The mean ORTO‐15 score of the participants was 37.3 ± 3.6, the mean EAT‐40 score was 20.0 ± 15.5, and the mean OCI‐R score was 25.9 ± 15.0. The ORTO‐15 scores of students from the Faculty of Medicine, the Faculty of Education, and those in formal education were found to be significantly lower (*p *< 0.05). EAT‐40 scores were significantly higher among students aged 20–22, those who perceived their socioeconomic status as poor, and smokers (*p* < 0.05). Participants who resided in a dormitory during their education and those who had quit smoking exhibited higher OCI‐R scores (*p* < 0.05). The ORTO‐15, EAT‐40, and OCI‐R scores of participants did not vary significantly by gender, parental education level, or alcohol use status (*p* > 0.05, Table 2).

**TABLE 2:** Distribution of ORTO‐15, EAT‐40, and OCI‐R scores according to the sociodemographic characteristics of the participants.

Sociodemographic characteristics	*n*	ORTO‐15, median (min–max)	EAT‐40, median (min–max)	OCI‐R, median (min–max)
Gender				
Female	645	37 (25–49)	15 (1–102)	25 (0–72)
Male	237	37 (27–46)	16 (2–102)	22 (0–72)
		*U* = 74,037.50, *p* = 0.473	*U* = 71,618.50, *p* = 0.151	*U* = 69,899.00, *p* = 0.051
Faculty				
Faculty of medicine	279	37 (27–46)	14 (2–102)	20 (0–72)*
Vocational school of health services	385	38 (26–49)^a^	15 (3–102)	24 (0–72)
Faculty of education	218	37 (25–48)^a^	17 (1–102)	28 (0–72)^a^
		*x* ^2^ = 10.239, ***p* = 0.006**	*x* ^2^ = 3.616, *p* = 0.164	*x* ^2^ = 15.598**, *p *< 0.001**
Grade				
First grade	335	38 (27–48)^a^	15 (3–102)^a^	23 (0–72)^a^
Second grade	273	37 (26–49)^a^	15 (1–102)^a^	27 (0–70)^a^
Third grade	116	37 (25–48)^a^	20 (1–94)^a^	28 (0–72)^a^
Fourth grade	55	37 (28–47)^a^	15 (5–70)	24 (4–65)
Fifth grade	30	37 (30–45)	13 (4–44)^a^	16 (3–52)^a^
Sixth grade	73	38 (27–44)^a^	14 (5–102)^a^	18 (0–72)^a^
		*x* ^2^ = 12.423, ***p* = 0.029**	*x* ^2^ = 14.752, ***p* = 0.011**	*x* ^2^ = 19.746, ***p* = 0.001**
Type of education				
Formal education	784	37 (25–49)	15 (1–102)	24 (0–72)
Secondary education	98	38 (29–46)	14 (3–75)	23 (0–67)
		*U* = 32,794.00, ***p* = 0.018**	*U* = 34,862.50, *p* = 0.135	*U* = 37,226.50, *p* = 0.617
Mother's education level				
Illiterate	141	37 (27–46)	15 (5–77)	23 (0–72)
Literate primary school	434	37 (25–49)	16 (1–102)	24 (0–72)
Middle school–high school	217	37 (28–48)	15 (2–92)	25 (0–67)
University and above	90	38 (29–46)	13 (5–77)	21 (0–72)
		*x* ^2^ = 7.415, *p* = 0.060	*x* ^2^ = 3.660, *p* = 0.301	*x* ^2^ = 3.673, *p* = 0.299
Father's education level				
Illiterate	19	36 (30–44)	13 (6–71)	22 (5–43)
Literate primary school	287	38 (25–47)	15 (1–102)	25 (0–72)
Middle school–high	345	37 (26–49)	16 (1–102)	25 (0–72)
University and above	231	37 (29–48)	15 (5–78)	23 (0–70)
		*x* ^2^ = 3.316, *p* = 0.345	*x* ^2^ = 0.972, *p* = 0.808	*x* ^2^ = 2.884, *p* = 0.410
Perception of socioeconomic situation				
Good	108	37 (27–48)	15 (4–74)	22 (0–65)
Middle	697	38 (25–49)	15 (1–102)^a^	24 (0–72)
Poor	77	37 (27–46)	18 (6–91)^a^	26 (4–72)
		*x* ^2^ = 3.265, *p* = 0.195	*x* ^2^ = 6.888, ***p* = 0.032**	*x* ^2^ = 2.360, *p* = 0.307
Place of residence during education				
With friends—Alone	76	37 (29–45)	14 (5–82)	22 (0–71)^a^
With family	485	37 (26–49)	15 (1–102)	24 (0–72)^a^
In the dormitory	321	37 (25–46)	16 (5–77)	26 (0–72)^a^
		*x* ^2^ = 1.117, *p* = 0.572	*x* ^2^ = 3.688, *p* = 0.158	*x* ^2^ = 7.661, ***p* = 0.022**
Place of residence before university				
City	637	37 (25–48)	15 (1–102)	24 (0–72)
Town	133	37 (29–49)	16 (1–82)	25 (0–68)
Village	112	38 (29–46)	15 (4–102)	21 (0–72)
		*x* ^2^ = 3.034, *p* = 0.219	*x* ^2^ = 0.323, *p* = 0.851	*x* ^2^ = 6.003, *p* = 0.050
Smoking				
Yes	133	38 (25–48)	18 (4–102)^a^	27 (0–68)^a^
Quit	55	37 (28–45)	16 (2–82)	28 (0–64)
No	694	37 (26–49)	15 (1–102)^a^	23 (0–72)^a^
		*x* ^2^ = 2.620, *p* = 0.270	*x* ^2^ = 9.621, ***p* = 0.008**	*x* ^2^ = 7.819, ***p* = 0.020**
Alcohol use				
Yes	60	37 (29–45)	17 (2–102)	26 (0–68)
Quit	38	38 (28–45)	17 (1–70)	27 (2–64)
No	784	37 (25–49)	15 (1–102)	24 (0–72)
		*x* ^2^ = 0.703, *p* = 0.704	*x* ^2^ = 2.072, *p* = 0.355	*x* ^2^ = 2.489, *p* = 0.288

^a^ Groups from which the difference originated (Bonferroni test).

EAT‐40 scores of the participants with chronic diseases and regular medication use were significantly higher (*p* < 0.05). The distribution of ORTO‐15, EAT‐40, and OCI‐R scores according to the health, nutrition, and physical activity characteristics of the participants is given in Table 3.

**TABLE 3:** Distribution of ORTO‐15, EAT‐40, OCI‐R scores according to health, nutrition, and physical activity characteristics of the participants.

Health, nutrition, and physical activity characteristics	n	ORTO‐15, median (min–max)	EAT‐40, median (min–max)	OCI‐R, median (min–max)
Chronic disease				
Yes	83	37 (29–48)	22 (5–77)	26 (0–66)
No	799	37 (25–49)	15 (1–102)	24 (0–72)
		*U* = 31,508.00, *p* = 0.453	*U* = 26,657.00, ***p* = 0.003**	*U* = 32,925.50*, p* = 0.916
Medication				
Yes	122	37 (28–45)	19 (2–91)	29 (0–68)
No	760	37 (25–49)	15 (1–102)	23 (0–72)
		*U* = 45,974.00*, p* = 0.882	*U* = 38,048.00, ***p* = 0.001**	*U* = 38,287.50, ***p* = 0.002**
Perception of health				
Good	443	38 (27–48)	14 (1–102)^a^	22 (0–72)^a^
Moderate	424	37 (26–49)	17 (5–102)^a^	25 (0–72)^a^
Poor	15	37 (25–46)	27 (8–77)^a^	32 (10–66)^a^
		*x* ^2^ = 1.807*, p* = 0.405	*x* ^2^ = 31.939, ***p* < 0.001**	*x* ^2^ = 13.838, ***p* = 0.001**
Body Mass Index (BMI)				
< 18.5 (Underweight)	128	38 (26–44)	15 (6–102)	23 (0–64)
18.5–24.9 (Normal weight)	589	37 (25–49)	15 (1–102)^a^	24 (0–72)
25.0–29.9 (Overweight)	147	37 (28–47)	18 (2–102)^a^	26 (0–72)
30.0 ≤ (Obese)	18	37 (32–44)	15 (10–68)	21 (3–63)
		*x* ^2^ = 0.571*, p* = 0.903	*x* ^2^ = 13.507, ***p* = 0.004**	*x* ^2^ = 2.434*, p* = 0.487
Regular breakfast				
Yes	479	38 (25–46)	15 (1–102)	24 (0–72)
No	403	37 (26–49)	16 (3–102)	24 (0–72)
		*U* = 93,874.00*, p* = 0.481	*U* = 90,281.00*, p* = 0.098	*U* = 91,523.00*, p* = 0.185
Perception of nutritional status				
Very good	29	37 (30–44)	24 (6–92)^a^	24 (4–60)
Good	621	37 (26–49)	14 (1–102)^a^	23 (0–72)^a^
Bad	211	38 (25–48)	18 (5–102)^a^	26 (0–72)^a^
Very bad	21	36 (27–48)	28 (11–77)^a^	36 (3–64)
		*x* ^2^ = 5.221*, p* = 0.156	*x* ^2^ = 43.477, ***p* < 0.001**	*x* ^2^ = 10.073, ***p* = 0.018**
Dieting status				
Yes	68	36 (25–44)	27 (6–102)	23 (0–70)
No	814	38 (26–49)	15 (1–102)	24 (0–72)
		***U* = 17,913.50*, p* < 0.001**	*U* = 14,798.00, ***p* < 0.001**	*U* = 27,169.00*, p* = 0.802
Regular physical activity				
Yes	239	37 (26–46)	17 (2–92)	23 (0–71)
No	643	37 (25–49)	15 (1–102)	25 (0–72)
		*U* = 74,106.50*, p* = 0.415	*U* = 66,783.00 ***, p* = 0.003**	*U* = 74,054.00*, p* = 0.408

^a^ Groups in which the difference occurred (Bonferroni test).

The ORTO‐15 scores of the participants were found to be negatively correlated with the EAT‐40, and there was a low positive correlation between OCI‐R and EAT‐40 scores (*p* < 0.05). No correlation was found between ORTO‐15 and OCI‐R (*p* > 0.05). There is a low positive correlation between participants' weight and age. There is a low positive correlation between EAT‐40 score and participants' weight. There is a low negative correlation between the OCI‐R score and age. There is a low negative correlation between the ORTO‐15 score and age. The correlation analysis of the variables is shown in Table 4.

**TABLE 4:** Correlation analysis of variables.

		Age	Weight	BMI	Number of family members	ORTO‐15	EAT‐40	OCI‐R
Age	*r*	1.000						
	*p*	—						
Weight	*r*	0.077	1.000					
	*p*	**0.022**	—					
BMI	*r*	0.119	0.851	1.000				
	*p*	**< 0.001**	**< 0.001**	—				
Number of family members	*r*	−0.069	−0.146	−0.106	1.000			
	*p*	**0.041**	**< 0.001**	**0.002**	**—**			
ORTO‐15	*r*	−0.087	−0.028	−0.046	−0.063	1.000		
	*p*	**0.010**	0.407	0.176	0.062	—		
EAT‐40	*r*	0.031	0.075	0.057	0.040	−0.288	1.000	
	*p*	0.359	**0.025**	0.091	0.238	**< 0.001**	—	
OCI‐R	*r*	−0.091	0.001	0.034	−0.002	−0.022	0.203	1.000
	*p*	**0.007**	0.974	0.308	0.950	0.516	**< 0.001**	—

*Note*: Spearman correlation analysis.

## 4 Discussion

In this study conducted among 882 participants, the relationship between ON and eating attitudes, behaviors, and obsessive–compulsive symptoms in university students who are prone to acquire unhealthy eating habits was examined.

It was determined that 14.7% of the participants had an orthorexic tendency. Studies have shown similar results to our study (Asil and Sürücüoğlu 2015; Sanlier et al. 2008). No significant difference in ORTO‐15 scores was found between genders in this study. This finding is in line with those in the literature (Brytek‐Matera et al. 2017; Demir and Savucu 2022; Farchakh et al. 2019; Plichta et al. 2019). However, there are also studies in the literature where orthorexic tendency is more common in male students (Culhacık and Durat 2017; Donini et al. 2004; Sanlier et al. 2008). The predominance of female participants in our study sample, comprising more than three‐quarters of the participants, may limit the ability to detect gender differences.

A low negative correlation was found between the participants' ages and ORTO‐15 scores. In a study conducted on sports science students, no relationship was found between age and ORTO‐15 (Demir and Savucu 2022). It can be thought that the reason for this finding was that the participants' ages were close to each other.

In our study, the ORTO‐15 scores of students studying at a health vocational school were found to be higher than those of students studying in other departments. This shows that they are more prone to normal eating behavior. Different from our study, there are studies showing that health department students are among the risk groups in terms of ON development (Ciarma and Mathew 2017; Fidan et al. 2010). As a matter of fact, in a similar study, no significant difference was reported between faculties in terms of ORTO‐15 scores (Demir and Savucu 2022; Farchakh et al. 2019; Sanlier et al. 2008). It can be thought that many etiological factors may be effective in the development of ON due to different results.

The participants' ORTO‐15 scores were high in the first and final years of study. Demir and Savucu (2022) found that ORTO‐15 scores did not change with students' grades. In this study, orthorexic tendency was more prevalent in participants who were on a diet. Arslantas et al. (2017) found that individuals who focused on optimal diet and calorie intake were more likely to have orthorexic tendencies. Considering that orthorexia means obsession with healthy eating, it is expected that participants who diet will have high orthorexic tendencies.

A quarter of the participants were found to be at risk of poor eating behavior. It was determined that 15.6% of the participants were at risk of impaired eating behavior. The mean EAT‐40 score of the participants was 20.0 ± 15.5, and there was no statistically significant difference between genders. In a similar study conducted among university students, no significant difference was found between genders (Sanlier et al. 2008; Yilmaz 2023). Research among university students showed that EDs are more common in females (Demir and Savucu 2022; Memon et al. 2012; Sanlier et al. 2008). Conversely, a different study found that men had a higher prevalence of inappropriate eating attitudes (Rouzitalab et al. 2015). Social disparities, changes in student distribution, and various departments may have affected the findings.

This study found that the EAT‐40 scores were higher among 20–22‐year‐olds and third‐grade students. A study by Asil and Sürücüoğlu (2015) identified a direct relationship between age and EAT‐40 scores. Conversely, Akdevelioglu and Yorusun (2019) noted a general decrease in the prevalence of ED as individuals transition from youth into adulthood. Various factors such as socioeconomic status, cultural background, and educational environment may influence the disparities shown in studies on the causes of ED.

Individuals who assessed their socioeconomic condition as low had higher EAT‐40 scores. Lower socioeconomic status correlates with increased challenges in obtaining adequate and high‐quality food, perhaps resulting in malnutrition (Thomas et al. 2002). Students who smoked had a higher EAT‐40 score. A study on adolescents indicated that the average EAT‐40 scores were higher in smokers, which aligns with our study (Harrison et al. 2020). In the literature, there are studies in which no correlation between smoking and EAT‐40 was found (Demir and Savucu 2022).

Our research revealed that students with chronic conditions who were taking medication had higher EAT‐40 scores. Research indicates that individuals with ED are more likely to develop chronic diseases (Maalouf et al. 2023). In addition, there were also studies that did not find a relationship between chronic disease and EAT‐40 (Demir and Savucu 2022). Students who perceived their health and nutritional status as poor had higher EAT‐40 scores. No significant correlation was found between BMI and EAT‐40 in our study. Demir and Savucu (2022) found no correlation between BMI and EAT‐40 in their study. There were also studies in which BMI and EAT‐40 average score showed a positive correlation (Rouzitalab et al. 2015). Students who followed a diet and participated in consistent physical activity had higher EAT‐40 scores. Demir and Savucu (2022) found that individuals who participated in physical activity had higher EAT‐40 scores, although the difference was not statistically significant.

Our study did not find a statistically significant difference in OCI‐R scores based on gender. The OCI‐R score averages were consistent with those reported in previous research (Davoudi et al. 2023; Varela Cunha et al. 2023). Students in the faculty of education and those in their third year of study had higher OCI‐R scores. In addition, those residing in dormitories had higher average OCI‐R scores compared to those living at home. The increased duties faced by students separated from their families may have led to a higher prevalence of OCD.

In our study, the average OCI‐R scores were higher among students who both smoked and had quit smoking. Liu et al. (2021) found that students who smoked had higher OCI‐R score averages compared to nonsmoking students in a similar study. Students with self‐reported poor health status and eating habits had higher OCI‐R scores. Students who reported regular medication use had higher OCI‐R scores. In a study similar to ours, it was discovered that individuals with chronic illnesses had higher occurrences of ED (Asil and Sürücüoğlu 2015). The continual usage of medication may have caused stress for the individual and heightened the likelihood of obsessive symptoms.

Participants' ORTO‐15 scores correlated negatively with their EAT‐40 scores. Our result was consistent with research conducted in different samples (Arslantas et al. 2017; Asil and Sürücüoğlu 2015; Culhacık and Durat 2017; Demir and Savucu 2022). Research has demonstrated that for every unit rise in the eating disorder scale score, the likelihood of being diagnosed with orthorexia increases by a factor of 1.07. This study identified a significant association between OCI‐R and EAT‐40 score means. While OCD and ED may appear distinct, they share many traits (Zagaria et al. 2022). Arusoğlu (2006) found that deterioration in eating attitudes and obsessive–compulsive symptoms were associated with orthorexic tendencies. EDs are characterized by a high rate of comorbidity with other psychiatric disorders, such as personality, mood, and anxiety disorders, especially OCD (Mandelli et al. 2020). Research has demonstrated a higher occurrence of OCD in individuals with ED compared to the general population (Ulfvebrand et al. 2015).

### 4.1 Limitations

As this study is cross‐sectional, it is unable to determine causality in the connections. Data were assessed by a questionnaire; therefore, the responses may not be conclusive. The ORTO‐15 is a screening tool for ON, which is sensitive to diet but has been demonstrated to fail in detecting the pathological stage of this condition.

## 5 Conclusion

Approximately 14% of the participants exhibit orthorexic tendencies and impaired eating behavior, with almost 50% of them being at risk for OCD. Additional research is required to explore potential risk factors for the strong connection between OCD, orthorexic tendencies, and ED. This investigation could help identify persons at risk and determine the need for preventive measures or targeted interventions. It is crucial to educate university students about nutrition, identify high‐risk groups for ED early, and implement preventive measures. Furthermore, the insufficient number of studies on ON in existing literature highlights the necessity for more extensive investigations on the topic.

## Author Contributions

**Mehtap Gomleksiz**: investigation, methodology, formal analysis, resources, writing – original draft, validation, data curation, supervision, conceptualization, writing – review and editing, project administration, visualization. **Ezgi Yarasir**: conceptualization, methodology, validation, formal analysis, resources, supervision, data curation, writing – review and editing, writing – original draft.

## Ethics Statement

Prior to the study, approval was obtained from the Non‐invasive Research Ethics Committee, including ethics committee permission (dated 27.09.2023, number 18586), institutional consent, and participant consent. The study was conducted in accordance with the Declaration of Helsinki.

## Peer Review

The peer review history for this article is available at https://publons.com/publon/10.1002/brb3.70583

## Data Availability

The data that support the findings of this study are available from the corresponding author upon reasonable request.
